# Dose-escalated radiotherapy for unresectable or locally recurrent pancreatic cancer: Dose volume analysis, toxicity and outcome of 28 consecutive patients

**DOI:** 10.1371/journal.pone.0186341

**Published:** 2017-10-12

**Authors:** Sebastian Zschaeck, Bibiana Blümke, Peter Wust, David Kaul, Marcus Bahra, Hanno Riess, Fritz Klein, Marianne Sinn, Uwe Pelzer, Volker Budach, Pirus Ghadjar

**Affiliations:** 1 Charité Universitätsmedizin Berlin, Department of Radiation Oncology, Berlin, Germany; 2 Charité Universitätsmedizin Berlin, Department of General, Visceral, and Transplantation Surgery, Berlin, Germany; 3 Charité Universitätsmedizin Berlin, Department of Hematology/Oncology/Tumorimmunology, Berlin, Germany; University of South Alabama Mitchell Cancer Institute, UNITED STATES

## Abstract

**Purpose:**

The role of radiotherapy for unresectable pancreatic cancer is controversial. A benefit of additional radiotherapy is supported by some observations. A dose-effect relationship was recently found by dose escalation employing image guided and intensity modulated radiotherapy.

**Methods:**

We retrospectively evaluated 28 consecutive patients, all with history of extensive prior therapies for unresectable locally advanced/ recurrent pancreatic cancer (LAPC/LRPC). Treatment was delivered by helical tomotherapy after daily position verification with computed tomography. Dose to the planned target volume (PTV) was 51 Gy, while the dose to the macroscopic tumor was escalated by a simultaneous integrated boost to a median cumulative dose of 66 Gy (60–66 Gy). Concomitant chemotherapy consisted mainly of capecitabine (n = 23).

**Results:**

10 of 28 patients presented acute toxicities > grade 2, one patient succumbed to gastrointestinal bleeding after treatment. No correlations of toxicities and dose volume histograms (DVH) of retrospectively delineated small bowel loops were observed, although average small bowel volume receiving ≥ 20 Gy was 374 ml. DVH analyses revealed a correlation of splenic parameters and acute toxicity: Vomiting, anorexia, dehydration, hematologic toxicity, fatigue, combined gastro-intestinal toxicity wit R-values between 0.392 and 0.561 (all p-values > 0.05). Only one patient developed late toxicities > grade 2. With an average follow-up time in surviving patients of 14 months median overall survival time was 19 months and median time to local recurrence 13 months. In 8 patients with available imaging of local recurrence: 5 in field recurrences, 2 marginal recurrences and one lymph node recurrence outside the high dose radiation field were observed. In univariate analysis only ΔCA-19-9 during radiotherapy was associated with local control (p = 0.029) and overall survival (p = 0.049).

**Conclusion:**

Dose escalated normo-fractionated radiotherapy for LAPC/LRPC seems feasible and suitable to prolong local control and in consequence long-term survival. However, in-field local progression is still frequently observed and possibilities to increase the local effectiveness should be evaluated. Exposure of the spleen was predictive for acute toxicity and should be further investigated.

## Introduction

Pancreatic cancer is an aggressive disease with unfavourable prognosis. Even for localized stages, the 5 year overall survival rate after curative resection and adjuvant chemotherapy is only around 20% [1,2]. For locally advanced stages (LAPC) unable to undergo surgery, the patients´ outcome is dismal and the goal of the treatment is survival prolongation and symptom control with a median overall survival (OS) between 5 to 11 months [3]. The prognosis for isolated locally recurrent pancreatic carcinomas is similar to initial LAPC with reported median overall survival around 6 months [4] if patients are not eligible for re-surgery [5,6].

The role of radiotherapy (RT) for LAPC (or LRPC) is controversial due to the high prevalence of distant metastases (long-term up to 70%) and the radiosensitivity of surrounding organs at risk (OAR) in the upper abdomen. As a consequence, the design of any study to evaluate the impact of radiotherapy is critical. First patient selection is crucial, because only in about 30% of patients outcome potentially could be improved by intensifying local treatment. Second the intolerances in the upper abdomen can mislead to prescribe ineffective radiotherapy schedules in order to avoid high toxicity.

In a couple of renowned studies radiochemotherapy (RCT) was evaluated, which combines sensitizing chemotherapy schemes with a more or less effective radiotherapy scheme. The sensitizing chemotherapy schedules were based on 5-FU or capecitabine and have only a limited systemic effect. The radiotherapy has been prescribed either as split-course schemes of 20 x 2 Gy until 40 Gy of low effectivity [1,7] or as standard schemes of 30 x 1.8 Gy until 54 Gy [8] or 30 x 2 Gy until 60 Gy [9]. These less effective RCT approaches are not able to improve a systemic chemotherapy [1,7] and are inferior, if they are applied front-line and replace or delay an effective systemic chemotherapy [1,9] because a large proportion of patients have already undetected dissemination and do not benefit from an early local treatment. Therefore, induction chemotherapy and re-staging was recommended to select patients for an additional consolidative RCT [10,11]. However even in a second-line approach (after 4 cycles of gemcitabine) RCT (54 Gy plus capecitabine) was not superior to a continuation of the gemcitabine chemotherapy in terms of overall survival [8]. Nevertheless a trend for improved progression-free survival was observed, probably caused by sustained local control.

In an ECOG-trial [12] initial RCT with gemcitabine (600 mg/sqm weekly plus 28 x 1.8 Gy until 50.4 Gy) was compared with gemcitabine 1000 mg/sqm weekly for six weeks, both arms followed by five cycles of gemcitabine at full dosage. Improved survival was found in the RCT arm. In this example, full systemic chemotherapy was only slightly reduced during radiotherapy and patients undergoing radiotherapy demonstrated improved overall survival.

On summary, the optimal usage of radiotherapy for LAPC is still under debate. Current data suggests that a consolidative approach after completing systemic treatment options and careful restaging might be reasonable. A positive role of post-operative radiotherapy was confirmed by a large retrospective analysis [13] which identified radiotherapy in addition to adjuvant chemotherapy as a favorable prognostic indicator for survival.

In a recent retrospective analysis focal radiation dose escalation seemed beneficial for patients with LAPC. Krishnan and colleagues reported on 47 patients that received dose escalation by a simultaneous integrated boost (SIB) and found improved overall survival when comparing these patients to a group of non-dose-escalated patients [14]. Due to the surrounding normal tissues dose-escalation within the upper abdomen can be challenging and the exact dose-response evaluation of LAPC as well as OAR is a matter of ongoing debate. Further information on dose-volume parameters and recurrence patterns is therefore provided by this study.

## Patients and methods

### Ethics

The data analysis for this study was approved by the Charité ethical review committee (EA1/236//16). Each participant provided written informed consent for publication of pseudonymized data.

### Patient characteristics

28 patients were treated between November 2012 and August 2016 for non-resectable LAPC (n = 15) or local recurrent pancreatic cancer after prior radical resection (n = 13). Radical resection consisted of pancreaticojejunostomy (n = 7), pancreatigogastrotomy (n = 5) or distal pancreatectomy (n = 1). The pancreatojejunostomy was performed in mattress positioning U-stitches (4–0 PDS with a MH1 needle) starting at the jejunal back wall, going from back to front, straight through the pancreatic remnant about 1 cm distal from the cut surface. Then the sutures were placed through the front wall of the jejunal loop. The suture than was brought straight through the pancreatic remnant.

The pancreatogastrotomy was performed also in mattress technique using U-stitches (4–0 PDS with a MH1 needle). After incision of the dorsal and ventral stomach the pancreatic remnant has been positioned into the dorsal stomach. The suture than was brought through the pancreas and the dorsal wall of the stomach. After that the ventral stomach was closed with sutures.

In case of distal pancreatectomy the pancreatic remnant closure after distal pancreatectomy has been performed using hand-sewn suturing of the pancreas without any anastomosis. All patients gave written informed consent and approval to conduct this study was obtained by the local Ethics Committee. Patients described here were heavily pre-treated, all patients received prior chemotherapy, either adjuvant after resection or in palliative intent or both. 7 out of 28 patients presented with distant metastases. These metastases were either regarded oligo-metastatic and underwent high dose radiotherapy (n = 3) or were in complete remission after palliative chemotherapy (n = 4). One additional patient who received high-dose neoadjuvant RCT was included for analysis of dose volume histograms and correlation with acute toxicity.

### Treatment, delineation and follow-up

Target and OAR delineation were performed using a contrast enhanced computed tomography (CT) and rigid registration of CT scans with with pancreatic protocol and oral contrast agent for bowel delineation and FDG-PET for tumor delineation whenever available. All patients were treated with helical tomotherapy with daily megavoltage CT (MVCT) position verification. Dose prescription was based on a prior pilot study that showed good treatment tolerability [15] and commonly consisted of 30 fractions of 1.7 Gray (Gy) to a total dose of 51 Gy to the planned treatment volume (PTV), which compromised the extended tumor region and regional lymphatic nodes (defined as clinical target volume = CTV with a safety margin of 5mm). CTV delineation was based on the risk of lymph node involvement proposed by Sun and colleagues [16]. For tumors of the pancreatic head posterior pancreatiocoduodenal, superior mesenteric and paraaortic nodes were included. For tumors of the pancreatic body or tail the lymphatic drainage around the splenic artery was partially included. Dose to the macroscopic tumor volume was escalated by a simultaneous integrated boost (SIB) with a single fraction dose of 2.2 Gy leading to a cumulative dose of 66 Gy. SIB dose prescription was reduced to 60 and 64.5 Gy in two patients due to OAR constraint infringing large macroscopic tumor volumes. The following OAR were contoured for clinical and investigational purposes: Stomach, small bowel loops, peritoneal cavity, spleen, kidneys, liver and spinal canal. Treatment planning and adherence to normal tissue constraints were based on peritoneal cavity while small bowel loops and spleen were retrospectively delineated and evaluated. Delineation was consensually performed by the same experienced radiation oncologist/radiologist (PW, SZ). 24 patients received simultaneous chemotherapy with either capecitabine (21 patients received 825 mg/m^2^ bi-daily, 2 patients received dose reduction, n = 23) or gemcitabine (600 mg/mq weekly, n = 1). All but one patient received > 90% of fractions by tomotherapy and the remaining one or two fractions by volumetric arc therapy, in the latter one only 15 fractions were delivered as planned and the remaining 15 fractions had to be re-planned on volumetric arc radiotherapy (VMAT) due to temporary breakdown of tomotherapy.

Toxicity was scored on a weekly basis based on Common Terminology Criteria for Adverse Events (CTCAE), version 4.0. Acute toxicity was defined as occurring during and up to 3 months after completion of radiotherapy (RT). Late toxicity was defined as occurring > 3 months after RT completion. Before and during the last week of or immediately after radiotherapy levels of serological tumor markers CEA and CA-19-9 were registered along with differential blood counts (the corresponding values are labeled with the suffixes preRT and postRT). For patients with normal Bilirubin levels percentual change of CA-19-9 during therapy was calculated as follows:

$$\Delta CA-19-9=\frac{CA-19-9preRT}{CA-19-9postRT}-1$$

Follow-up visits were usually performed at least each three months after terminating RT. Commonly follow-up included CT scans of the upper abdomen and lung and/ or abdominal sonography. In case of clinical uncertainties patients were referred for whole body FDG-PET-CT. Loco-regional tumor response at the first follow-up visit, 3 months after terminating RT was evaluated using response evaluation criteria in solid tumors (RECIST), version 1.1.

### Dose-volume and imaging analyses

Tomotherapy treatment plans of patients receiving > 90% fractions on tomotherapy were imported into ARIA® treatment planning software (Varian, Palo Alto, CA, USA) and dose volume histograms (DVH) for all target volumes and OAR were calculated and exported for further analysis. Maximal, median and average dose was calculated for all OAR, furthermore volumes receiving 5 to 60 Gy were calculated in 5 Gy steps for the following OAR: Small bowel loops (volume in ml), stomach (in % of total volume) and spleen (in % of total volume).

In case of local recurrence during follow-up, imaging of local recurrence (CT or FDG-PET-CT) was rigidly co-registered to the treatment plan. After delineation of local recurrence the spatial distribution of recurrence concerning RT dose was performed. Since size of recurrences differed substantially, the volume of recurrence was iso-volumteric reduced to an origin of recurrence volume measuring 4–5 ml. Dose parameters to this volume and to the whole recurrence volume were analysed to distinguish between in field recurrences, marginal recurrences and out of field recurrences.

### Statistical analyses

All statistical analyses were performed using IBM® SPSS®, version 24 (IBM Corporation, Armonk, NY, USA). Correlation of acute and late toxicity with DVH was performed using non-parametric Spearman analysis. CTCAE scores of acute gastro-intestinal toxicities (nausea, vomiting, diarrhea, constipation, anorexia, dehydration, abdominal pain, hemorrhage, dyspepsia, gastro-esophageal reflux) were additionally summed up as combined gastro-intestinal toxicity.

Oncological endpoints of the study were local control (LC), overall survival (OS), occurrence of distant metastasis (DM, only for patients without metastases prior to RT) and progression free survival (PFS). The impact of potential prognostic and clinical variables on the endpoints were evaluated using the univariate Cox-regression model, corresponding survival curves were estimated by the Kaplan-Meier method, starting on the first day of radiotherapy.

## Results

Median patient age was 66 years (range: 45 to 77 years). In case of local recurrent disease after radical surgery median time between surgery and start of radiotherapy was 541 days (range: 43 to 2297 days). Table 1 summarizes patient characteristics.

**Table 1:** Patient characteristics.

**Gender**	
Male	19
Female	9
**Tumor location**	
Pancreas head	19
Pancreas body	7
Pancreas tail	2
**Initial UICC stage**	
I	2
IIA	6
IIB	6
III	9
IV	5
**Tumor markers (median value and range)**	
Initial CA-19-9 (U/ml)	215 (0.5–4926)
Initial CEA (ng/ml)	2.85 (1.1–12.9)
CA-19-9 (U/ml) before radiotherapy	122.7 (9.4–3474)
CEA before radiotherapy	4.1 (0.9–57.2)
CA-19-9 (U/ml) after radiotherapy	85 (0.5–5146)
CEA after radiotherapy	3.85 (1.1–49.5)
Prior neoadjuvant Chemotherapy	
None	26
Gemcitabine/ Nab-Paclitaxel	2
**Prior palliative Chemotherapy**	
None	6
FOLFIRINOX	13
Gemcitabine/ Nab-Paclitaxel	9
**Prior surgery**	
Pylorus preserving Pancreatico-duodenectomy	9
Whipple procedure	2
Distal Pancreatectomy	2
R0 resection	7
R1 resection	5
R2 resection	1
none	15

Treatment was well tolerated in most patients. Ten of 28 patients presented acute toxicities > grade 2, most frequently weight loss was observed. One patient succumbed to gastrointestinal bleeding two weeks after end of treatment. This patient already presented severe anemia and fatigue before initiation of radiotherapy, probably due to undetected bloody oozing. Another patient developed controllable mild gastrointestinal bleeding during mid-treatment which was most probably tumor related. Table 2 summarizes the observed toxicities of RT. Evaluation of DVH revealed a weak correlation of intestine volume receiving ≥ 50 Gy and nausea (R = 0.403; p = 0.034). Also a significant correlation between anemia and various DVH parameters of the stomach (Average dose, median dose, V10%, 20%, 30%, 40%) was observed (R between 0.398 and 0.434; p between 0.024 and 0.04). Table 3 shows stomach and small intestine volumes receiving 10, 20, 30 and 40 Gy in individual patients and average values.

**Table 2:** Observed acute radiation induced toxicities.

**Nausea**	
Grade 0	7
Grade 1	15
Grade 2	5
Grade 3	1
**Vomiting**	
Grade 0	24
Grade 1	4
**Diarrhea**	
Grade 0	12
Grade 1	12
Grade 2	4
**Constipation**	
Grade 0	26
Grade 1	1
Grade 2	1
**Anorexia**	
Grade 0	7
Grade 1	7
Grade 2	6
≥ Grade 3	8
**Dehydration**	
Grade 0	24
Grade 1	1
Grade 2	3
**Abdominal pain**	
Grade 0	14
Grade 1	5
Grade 2	7
Grade 3	2
**Gastrointestinal bleeding**	
Grade 0	26
≥ Grade 3	2
**Dyspepsia**	
Grade 0	26
Grade 1	1
Grade 2	1
**Gastroesophageal reflux**	
Grade 0	26
Grade 2	2
**Fatigue**	
Grade 0	8
Grade 1	6
Grade 2	10
Grade 3	3
n.a.	1
**Hematologic toxicity**	
Grade 0	24
Grade 1	3
n.a.	1

Toxicities scored according to CTCAE 4.0

**Table 3:** Stomach and small bowel dose volume histograms.

Pat.	Stomach				bowel loops			
	V10 (%)	V20 (%)	V30 (%)	V40 (%)	V10 (ml)	V20 (ml)	V30 (ml)	V40 (ml)
**1**	97	80	66	58	820	368	149	69
**2**	28	21	12	5	661	212	96	32
**3**	45	24	5	1	531	327	92	31
**4**	81	47	14	4	816	414	147	61
**5**	83	71	57	39	721	544	289	79
**6**	62	52	28	2	743	320	146	60
**7**	16	10	6	2	1200	445	130	39
**8**	31	13	4	1	648	310	100	19
**9**	60	26	8	3	505	391	281	135
**10**	61	14	4	1	972	330	188	104
**11**	38	20	1	0	780	516	287	166
**12**	38	29	17	5	924	631	179	62
**13**	14	12	11	9	780	409	169	60
**14**	34	24	4	0	660	402	142	44
**15**	73	46	27	11	1026	616	240	105
**16**	64	37	14	5	642	356	129	32
**17**	91	84	72	45	606	392	167	83
**18**	37	8	1	0	477	196	56	10
**19**	53	32	7	1	248	144	77	31
**20**	55	41	11	1	604	302	91	23
**21**	100	75	36	16	2153	814	305	121
**22**	18	5	1	0	1218	525	102	23
**23**	57	32	11	2	736	261	77	18
**24**	19	13	7	2	803	425	171	47
**25**	39	21	10	1	711	251	79	43
**26**	50	34	15	2	485	158	33	3
**27**	54	27	9	1	723	310	109	26
**28**	98	73	46	23	287	114	40	11
**Avg.**	**53%**	**35%**	**18%**	**9%**	**767 ml**	**374 ml**	**145 ml**	**55 ml**

Volumes of the stomach (in %) and of bowel loops (in ml) receiving 10, 20, 30 and 40 Gy (V10, V20, V30, V40) in individual patients (Pat.) and on average (Avg.).

Dose volume parameters of the spleen showed the highest correlation with several toxicities (vomiting, anorexia, dehydration, hematologic toxicity, fatigue, combined gastro-intestinal toxicity) wit R-values between 0.392 and 0.561 (all p-values significant), Table 4 shows observed toxicities and spleen dose-volume parameters. No significant correlations between patient characteristics, clinical parameters or PTV volumes and toxicity were observed. S1 Table lists individual DVH parameters for selected OAR. Higher grade (> grade 2) late toxicity was only observed in one patient, who presented substantial weight loss. However this patient suffered from local and systemic relapse, therefore an association of these symptoms with radiation therapy is doubtful.

**Table 4:** Dose volume parameters of the spleen (individual maximal, average and median dose and spleen volumes receiving 10 to 40 Gy) and correlation to acute toxicities.

Toxicity		Spleen D_Max_	Spleen D_Mean_	Spleen D_Median_	Spleen V_10_	Spleen V_20_	Spleen V_30_	Spleen V_40_
**Vomiting**	**R**	0.242	0.061	0.000	0.166	0.076	0.219	**0.45**
	**p**	0.244	0.774	1.000	0.427	0.712	0.292	**0.024**
**Diarrhea**	**R**	0.344	**.440**	**.419**	**.445**	0.270	**.430**	0.338
	**p**	0.100	**0.031**	**0.042**	**0.029**	0.192	**0.036**	0.106
**Anorexia/Loss of Appetite**	**R**	**0.561**	0.387	0.326	**0.437**	**0.447**	0.299	0.312
	**p**	**0.004**	0.056	0.112	**0.029**	**0.022**	0.147	0.129
**Hematologic toxicity**	**R**	0.173	**0.446**	**0.446**	0.391	0.258	0.351	0.154
	**p**	0.419	**0.029**	**0.029**	0.059	0.214	0.092	0.472
**Combined GI**	**R**	**0.498**	**0.437**	0.379	**0.458**	**0.392**	0.331	**0.526**
	**p**	**0.011**	**0.029**	0.062	**0.021**	**0.048**	0.106	**0.007**
**Fatigue**	**R**	0.152	-0.025	-0.043	-0.073	0.146	0.259	**0.408**
	**p**	0.478	0.906	0.843	0.735	0.487	0.221	**0.048**

D_Max_ = Maximal splenic dose, D_Mean_ = Average splenic dose, D_Median_ = median splenic dose. V_10_ –V_40_ = splenic volumes (in % of the whole organ) receiving 10 to 40 Gy radiation dose. R = correlation coefficient, p = p-value, significant p-values < 0.05 in bold,.

Local response evaluation three months after completing RT was available for 24 patients and revealed stable disease in 14 cases, partial remission in 9 cases and one case of progressive disease.

**Fig 1:**
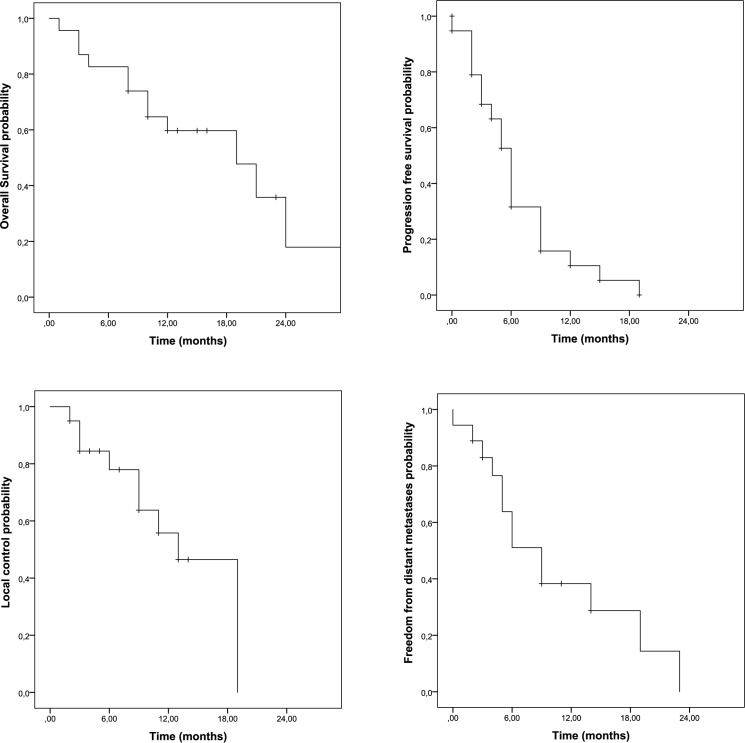
Kaplan-Meier curves showing overall survival and progression free survival probability (above) and freedom from local recurrence or distant metastases rate (below).

**Fig 2:**
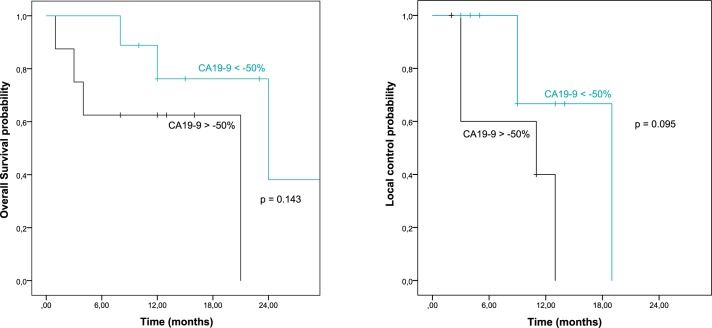
Dichotomization of patients regarding CA-19-9 plasma marker response and association with overall survival and local control probability.

With an average follow-up time of 14 months in surviving patients, median OS time was 19 months, median time to local recurrence was 13 months. Fig 1 depicts the Kaplan-Meier curves for OS, PFS, LC and DM. In univariate analysis only the ΔCA-19-9 response during RCT was significantly associated with outcome (OS, p = 0.049; LC, p = 0.029;). All other factors, including age, tumor location, distant metastases prior to RT and splenic dose parameters did not show any association with OS. Results of univariate analyses are summarized in S2 Table. Dichotomization of ΔCA-19-9 (decrease of 50% or more versus decrease of less than 50% or increase during RCT) revealed a trend for better local control in patients with more pronounced CA-19-9 responses (p = 0.095) as shown in Fig 2.

Sectional imaging information of patients with local recurrences was available in 8 of 11 cases. 5 patients developed in field recurrences, 2 patients marginal recurrences and one patient developed a lymphnode recurrence outside the RT field. Table 5 shows doses to the recurrent tumor volumes and corresponding GTV volumes.

**Table 5:** Recurrence patterns.

GTV volume	Median dose	Median dose	Minimal dose	Time to failure*	Failure
	recurrence	OOR	OOR		
31.4	43.6	44.2	30.0	13	marginal
123.3	65.0	65.8	39.1	4	in field
45.3	6.3	8.3	1.6	9	out of field
350.0	64.9	66.4	64.0	2	in field
33.5	65.2	66.7	61.6	11	in field
40.5	58.8	65.8	39.9	9	in field
57.1	36.1	38.0	26.5	13	marginal
65.2	57.0	57.5	51.6	3	In field

GTV volume = irradiated gross tumor volume in ml. OOR = origin of recurrence (isovolumetric shrunk recurrence volume). All doses in Gray.

*given in months, measured from start of radiotherapy.

## Discussion

Here we report our first experience with daily image guided dose-escalated RT for LAPC. Compared to the 47 dose escalated patients described by Krishnan and colleagues the median OS observed in our study was similar (17.8 months versus 19 months in our study) although 25% (7 of 28) of the patients described here presented with distant metastases. Additionally no clear correlation between classical OAR DVH and toxicity was found, in contrast several significant associations of spleen DVHs and acute toxicities were observed. Furthermore CA-19-9 response during RCT could be identified as a potential novel biomarker to select patients that most likely benefit from focal dose-escalated RT.

Regarding outcome after high-dose radiotherapy similar results were reported by Chung and colleagues: In a retrospective analysis of 152 patients receiving radiochemotherapy with 61 Gy or more the median overall survival time was 21.9 months [17]. In a phase-I/II dose escalation trial Ben-Josef and colleagues reported a median overall survival time of 14.8 months with most patients of the study receiving 55 Gy prescribed dose [18]. Another Phase-II study with induction chemotherapy followed by radiochemotherapy up to 59.4 Gy total radiation dose reported a median overall survival time of 14 months in unresectable patients [19]. Despite further dose escalation, permanent local tumor control is still unsatisfactory with in-field recurrences in most cases. Obviously, the radiation dosage is still too low. Considering the described advantage of dose escalation [14,17] a further increase of dose might be required to warrant local control. Therefore, uncertainties causing geographical misses must be minimized to enable further dose escalation and improve dose coverage while adjacent OARs are still sufficiently spared. One error source could be intra-fractional variation due to respiratory motion [20,21]. GTV was delineated using FDG-PET whenever available. Due to the long acquisition time PET imaging contains some information on respiratory motion, however even 3D PET underestimates respiratory motion as a recent publication on 4D PET for pancreatic cancer showed [22]. In our study we instructed the patients to breathe shallow during planning CT as well as during dose delivery. The instantaneous equilibrium position of the tumor was adjusted via MCVT exploiting soft tissue contrast and stents in situ as far as possible. Further evaluation based on 4D-CT and methods to determine and minimize intrafractional displacements are required.

If further dose escalation in the upper abdomen is indispensible, small bowel and in particular the duodenum are limiting the magnitude and coverage of tumor dose. Published data on OAR constraints and toxicity show a large heterogeneity. For pancreatic cancer Kelly described a correlation of V_55Gy_ within the duodenum and gastro-intestinal toxicity [23], however in a small cohort of patients treated with hypofractionated stereotactic RT duodenal DVHs only correlated to histomorphological alterations but not to clinical scored toxicity [24]. The best available data on small intestine constraints from QUANTEC recommends that the dose to the small bowel receiving ≥ 45 Gy should not exceed 200 ml, if the entire peritoneal space is delineated. This constraint was always adhered to for treatment planning. For delineation of small bowel loops QUANTEC recommends that the dose to the small bowel receiving ≥ 15 Gy should not exceed 120ml. Retrospective additional delineation of small bowel loops showed excessive doses compared to the latter QUANTEC constraints, as shown in Table 3, with relatively good tolerability. The DVH-values of intestine in our study were not associated with relevant intestinal acute toxicity. Furthermore addition of chemotherapy or previous surgery is known for an additional, though in its magnitude unknown, contribution to toxicity [25]. Type of surgery and anastomosis may have an additional effect on radiation toxicity. However surgical procedures were relatively homogeneous as most patients received pylorus preserving pancreaticoduodenectomy and a recent Cochrane analysis showed that postoperative comorbidities do not differ dramatically between classical Whipple procedure and pylorus preserving surgery [26].

In all patients higher volumes of the intestine were exposed to 30 Gy and 20 Gy (Table 3). However, QUANTEC data is mainly based on DVH analyses of rectal and cervical cancer patients. Explicit DVH data for irradiation of pancreatic cancers is sparse. Jin and colleagues analysed DVHs of 20 patients treated for pancreatic cancers and found similar, comparatively low rates of higher grade toxicity. Additionally an advantage of VMAT compared to 3D conformal RT or intensity modulated RT (IMRT) regarding acute toxicities was described by several groups [27,28]. These findings are in contrast to the Phase-I/II study on radiation dose escalation reported by Ben-Josef and colleagues: They observed an increase of dose limiting toxicities with radiation doses higher than 55 Gy [18]. However no information on daily image guidance was reported, which may be an important difference to our and other studies who reported the tolerability of higher radiation doses. The missing correlation of small bowel dose volume parameters and acute toxicity when using modern RT techniques was also described by another group who investigated preoperative RCT for rectal cancer [29].

Besides the absent association of classical OAR dosimetric factors and toxicity, we found instead several associations between DVH parameters of the spleen and toxicity. Traditionally the spleen is an ignored organ in RT, although with the onset of radio-immunotherapy it currently raised some interest. Trip found dose-dependend volumetric long-term alterations of the spleen in patients treated for gastric cancers [30]. In irradiated pancreatic cancer patients splenic DVH parameters were associated with development of severe lymphopenia after RT, the latter one being prognostic for patient survival [31]. No information about a correlation of splenic DVHs and other than hematologic toxicities were reported in that publication. No association of splenic dose parameters and oncological outcome parameters (LC, OS, DM) was observed in our study upon univariate testing. The explanation for the observed association between splenic DVH-parameters and toxicity remains unresolved and it is unlikely that all observed side effects are attributable to the spleen doses. The spleen might most likely be a surrogate organ to quantify the dose exposure specifically to the upper abdomen, which is correlated with general complaints such as anorexia and fatigue. Even when patients are carefully instructed about timing of ingestion, stomach dose volume parameters still fluctuate largely, which may be an explanation why no strong correlations were observed when evaluationg DVH parameters of the stomach. Splenic DVH parameters should be further evaluated and validated.

Pre-therapeutic CA-19-9 levels are known prognostic factors for OS and PFS in patients treated with RCT for LAPC, however the role of pre-therapeutic levels in case of radical surgery is controversial [32–34]. In this cohort of patients we were not able to confirm a prognostic role of initial CA-19-9, however patients described here had relatively high CA-19-9 levels as only 4 of 22 patients would have belonged to the favourable prognostic group with CA-19-9 lower or equal 90 U/ml described by Vainshtein [32]. However individual CA-19-9 decrease was found to be significantly associated with OS and LC. Our findings are in line with another publication: Koom and colleagues identified CA-19-9 decrease of less than 40% as a strong negative prognostic factor for patients undergoing RCT for pancreatic cancer [35], however the latter study measured the second CA-19-9 value one to three months after RT, while our study measured CA-19-9 values during the last week or immediately after radiotherapy, when therapeutic alterations of the treatment regime would still be potentially feasible.

Our study has several limitations: First of all its retrospective nature, although patients were treated within an internal standardized protocol and all consecutive patients were evaluated. Second the heterogeneity of patients: Patients with distant metastases as well as patients with non-resectable or local recurrent cancer were included. However, the latter two groups are very similar regarding prognosis and presence of distant metastases prior to RT was not associated with inferior outcome upon univariate testing. Another limitation regards the relatively low patient number, therefore no multivariate analysis was performed to avoid statistical overfitting, furthermore our findings should be seen as hypothesis generating. In this regard high-dose daily image-guided normo-fractionated RCT for LAPC seems to be relatively safe, while established OAR restrictions were not associated with acute toxicity. Our findings indicate that the spleen should potentially be included as an OAR for high-dose RT of the upper abdomen. Furthermore, CA-19-9 response during irradiation might be useful for treatment stratification. Last but not least the low LC rate observed in our study indicates that RT in this setting needs further intensification: Either by the use of novel systemic agents [36,37], by the addition of hyperthermia to RCT [38], and particularly by selectively increasing the radiation dose to morphological defined high-risk regions, which requires improved image guidance and compensation of organ motion during dose delivery. The proof of principle that high radiation doses within a small volume are well tolerable could also be used for future trials in unresectable or borderline resectable pancreatic cancer. Instead of boosting the whole macroscopic tumor, a boost to infiltrated vessels might be better tolerated and consecutive surgery could substantially improve local control [39,40].

## Supporting information

S1 Table Representative dose volume parameters of the stomach and intestine and their correlation with acute toxicity. (DOCX)

S2 Table Univariate analyses for overall survival and patient variables. 95% CI = 95 percent confidence interval. Local recurrence = local recurrent versus initially local advanced pancreatic cancer. Distant metastases = distant metastasis before radiotherapy.
(DOCX)
