# Supplementary material for: Dose-escalated radiotherapy for unresectable or locally recurrent pancreatic cancer: Dose volume analysis, toxicity and outcome of 28 consecutive patients
Source: PLoS One. 2017 Oct 12;12(10):e0186341. doi: 10.1371/journal.pone.0186341 (PMC5638513; doi:10.1371/journal.pone.0186341)
Supplement: S1 Table — (DOCX) [file pone.0186341.s001.docx]

| **Toxicity** |  | **Stomach**  **Average dose** | **Stomach**  **Median**  **dose** | **Stomach**  **V10 %** | **Stomach**  **V20 %** | **Stomach**  **V30 %** | **Stomach**  **V40 %** | **Intestine**  **Maximum dose** | **Intestine**  **Average dose** | **Intestine**  **Median**  **dose** | **Intestine**  **V10 ml** | **Intestine**  **V20 ml** | **Intestine**  **V30 ml** | **Intestine**  **V40 ml** | **Intestine**  **V50 ml** |
| --- | --- | --- | --- | --- | --- | --- | --- | --- | --- | --- | --- | --- | --- | --- | --- |
| **Nausea** | **R** | 0,164 | 0,167 | 0,176 | 0,205 | 0,116 | 0,069 | 0,308 | 0,045 | -0,033 | 0,094 | 0,086 | 0,136 | 0,304 | **0,403** |
|  | **p** | 0,405 | 0,394 | 0,371 | 0,296 | 0,556 | 0,726 | 0,111 | 0,822 | 0,867 | 0,634 | 0,665 | 0,492 | 0,116 | **0,034** |
|  | **n** | 28 | 28 | 28 | 28 | 28 | 28 | 28 | 28 | 28 | 28 | 28 | 28 | 28 | 28 |
| **Vomiting** | **R** | 0,088 | 0,114 | 0,139 | 0,152 | -0,025 | -0,088 | 0,101 | 0,126 | 0,076 | -0,088 | 0,152 | 0,215 | 0,215 | 0,152 |
|  | **p** | 0,654 | 0,565 | 0,481 | 0,441 | 0,898 | 0,654 | 0,609 | 0,522 | 0,701 | 0,654 | 0,441 | 0,272 | 0,272 | 0,441 |
|  | **n** | 28 | 28 | 28 | 28 | 28 | 28 | 28 | 28 | 28 | 28 | 28 | 28 | 28 | 28 |
| **Diarrhea** | **R** | -0,077 | 0,056 | 0,103 | -0,082 | -0,149 | 0,032 | -0,106 | 0,155 | 0,100 | 0,071 | 0,236 | 0,324 | 0,149 | -0,064 |
|  | **p** | 0,702 | 0,782 | 0,609 | 0,685 | 0,458 | 0,874 | 0,598 | 0,441 | 0,618 | 0,724 | 0,236 | 0,100 | 0,457 | 0,753 |
|  | **n** | 27 | 27 | 27 | 27 | 27 | 27 | 27 | 27 | 27 | 27 | 27 | 27 | 27 | 27 |
| **Constipation** | **R** | -0,253 | -0,148 | -0,180 | -0,323 | **-0,453** | **-0,454** | 0,069 | 0,122 | 0,103 | -0,134 | -0,042 | -0,003 | 0,035 | 0,035 |
|  | **p** | 0,204 | 0,462 | 0,369 | 0,101 | **0,018** | **0,017** | 0,734 | 0,543 | 0,607 | 0,504 | 0,837 | 0,989 | 0,863 | 0,863 |
|  | **n** | 27 | 27 | 27 | 27 | 27 | 27 | 27 | 27 | 27 | 27 | 27 | 27 | 27 | 27 |
| **Anorexia/Loss of Appetite** | **R** | 0,069 | 0,189 | 0,100 | 0,039 | -0,076 | -0,029 | 0,365 | 0,129 | 0,107 | 0,032 | 0,043 | 0,081 | 0,060 | 0,088 |
|  | **p** | 0,727 | 0,336 | 0,612 | 0,845 | 0,700 | 0,885 | 0,056 | 0,512 | 0,589 | 0,873 | 0,827 | 0,682 | 0,763 | 0,655 |
|  | **n** | 28 | 28 | 28 | 28 | 28 | 28 | 28 | 28 | 28 | 28 | 28 | 28 | 28 | 28 |
| **Dehydration** | **R** | 0,201 | 0,214 | 0,245 | 0,186 | 0,063 | 0,000 | 0,003 | 0,077 | -0,034 | -0,026 | 0,064 | 0,196 | 0,144 | 0,047 |
|  | **p** | 0,306 | 0,274 | 0,208 | 0,343 | 0,752 | 0,998 | 0,989 | 0,695 | 0,863 | 0,897 | 0,747 | 0,317 | 0,464 | 0,811 |
|  | **n** | 28 | 28 | 28 | 28 | 28 | 28 | 28 | 28 | 28 | 28 | 28 | 28 | 28 | 28 |
| **Adbominal Pain** | **R** | 0,248 | 0,243 | 0,179 | 0,205 | 0,182 | 0,115 | -0,091 | -0,028 | 0,016 | 0,024 | 0,159 | 0,023 | -0,051 | -0,071 |
|  | **p** | 0,204 | 0,213 | 0,361 | 0,296 | 0,355 | 0,558 | 0,643 | 0,888 | 0,937 | 0,904 | 0,418 | 0,907 | 0,798 | 0,718 |
|  | **n** | 28 | 28 | 28 | 28 | 28 | 28 | 28 | 28 | 28 | 28 | 28 | 28 | 28 | 28 |
| **GI hemorrhage** | **R** | -0,033 | 0,002 | -0,047 | -0,083 | -0,014 | 0,004 | 0,108 | 0,127 | 0,214 | 0,214 | 0,343 | 0,145 | -0,027 | -0,018 |
|  | **p** | 0,867 | 0,990 | 0,812 | 0,676 | 0,943 | 0,985 | 0,585 | 0,520 | 0,273 | 0,275 | 0,074 | 0,463 | 0,892 | 0,926 |
|  | **n** | 28 | 28 | 28 | 28 | 28 | 28 | 28 | 28 | 28 | 28 | 28 | 28 | 28 | 28 |
| **Dyspepsia** | **R** | 0,285 | 0,266 | 0,231 | 0,231 | 0,192 | 0,211 | 0,321 | -0,035 | 0,039 | 0,001 | 0,024 | 0,003 | -0,085 | -0,132 |
|  | **p** | 0,150 | 0,180 | 0,246 | 0,246 | 0,337 | 0,291 | 0,102 | 0,863 | 0,847 | 0,995 | 0,905 | 0,989 | 0,675 | 0,513 |
|  | **n** | 27 | 27 | 27 | 27 | 27 | 27 | 27 | 27 | 27 | 27 | 27 | 27 | 27 | 27 |
| **Gastroesophageal reflux disease** | **R** | -0,018 | -0,109 | -0,109 | -0,036 | 0,054 | 0,145 | 0,272 | -0,254 | -0,236 | -0,036 | -0,327 | -0,309 | -0,254 | -0,091 |
|  | **p** | 0,928 | 0,589 | 0,589 | 0,857 | 0,787 | 0,470 | 0,169 | 0,201 | 0,236 | 0,857 | 0,096 | 0,117 | 0,201 | 0,652 |
|  | **n** | 27 | 27 | 27 | 27 | 27 | 27 | 27 | 27 | 27 | 27 | 27 | 27 | 27 | 27 |
| **Hematologic toxicity** | **R** | 0,227 | 0,318 | 0,242 | 0,151 | 0,151 | 0,182 | 0,166 | 0,227 | 0,197 | -0,091 | -0,015 | 0,045 | 0,015 | -0,045 |
|  | **p** | 0,255 | 0,106 | 0,224 | 0,451 | 0,451 | 0,365 | 0,407 | 0,255 | 0,325 | 0,652 | 0,940 | 0,822 | 0,940 | 0,822 |
|  | **n** | 27 | 27 | 27 | 27 | 27 | 27 | 27 | 27 | 27 | 27 | 27 | 27 | 27 | 27 |
| **Anemia** | **R** | **0,418** | **0,418** | **0,398** | **0,398** | **0,417** | **0,434** | 0,367 | **0,4** | 0,367 | 0,103 | 0,188 | 0,278 | 0,237 | 0,157 |
|  | **p** | **0,030** | **0,030** | **0,040** | **0,040** | **0,031** | **0,024** | 0,060 | **0,038** | 0,060 | 0,607 | 0,347 | 0,160 | 0,235 | 0,433 |
|  | **n** | 27 | 27 | 27 | 27 | 27 | 27 | 27 | 27 | 27 | 27 | 27 | 27 | 27 | 27 |
| **combined GI** | **R** | 0,057 | 0,146 | 0,093 | 0,008 | -0,103 | -0,060 | 0,330 | 0,154 | 0,141 | 0,194 | 0,306 | 0,231 | 0,114 | 0,117 |
|  | **p** | 0,774 | 0,459 | 0,637 | 0,968 | 0,600 | 0,763 | 0,087 | 0,434 | 0,474 | 0,323 | 0,113 | 0,238 | 0,562 | 0,554 |
|  | **n** | 28 | 28 | 28 | 28 | 28 | 28 | 28 | 28 | 28 | 28 | 28 | 28 | 28 | 28 |
| **Fatigue** | **R** | 0,099 | 0,146 | 0,024 | 0,142 | 0,232 | 0,169 | -0,038 | 0,141 | 0,130 | -0,015 | 0,231 | 0,227 | 0,146 | 0,066 |
|  | **p** | 0,622 | 0,468 | 0,906 | 0,481 | 0,245 | 0,399 | 0,851 | 0,482 | 0,517 | 0,942 | 0,246 | 0,255 | 0,468 | 0,742 |
|  | **n** | 27 | 27 | 27 | 27 | 27 | 27 | 27 | 27 | 27 | 27 | 27 | 27 | 27 | 27 |

**Supplementary table 1:** Representative dose volume parameters of the stomach and intestine and their correlation with acute toxicity.
