# Supplementary material for: Dose-escalated radiotherapy for unresectable or locally recurrent pancreatic cancer: Dose volume analysis, toxicity and outcome of 28 consecutive patients
Source: PLoS One. 2017 Oct 12;12(10):e0186341. doi: 10.1371/journal.pone.0186341 (PMC5638513; doi:10.1371/journal.pone.0186341)
Supplement: S2 Table — 95% CI = 95 percent confidence interval. Local recurrence = local recurrent versus initially local advanced pancreatic cancer. Distant metastases = distant metastasis before radiotherapy. (DOCX) [file pone.0186341.s002.docx]

| **Variable** | **Hazard Ratio (95% CI)** | **p-Value** |
| --- | --- | --- |
| Karnofsky score | 0.99 (0.94 – 1.05) | 0.86 |
| Age | 1.00 (0.95 – 1.05) | 0.97 |
| Local recurrence | 1.61 (0.51 -5.11) | 0.42 |
| Tumor location | 0.82 (0.39 – 1.73) | 0.60 |
| Initial CA-19-9 | 1.00 (1.00 – 1.01) | 0.19 |
| Initial CEA | 0.94 (0.77 – 1.14) | 0.52 |
| Distant metastases | 1.39 (0.29 – 6.72) | 0.68 |
| CA-19-9 response in % | 1.01 (1.00 – 1.01) | 0.049 |

**Supplementary table 2:** Univariate analyses for overall survival and patient variables. 95% CI = 95 percent confidence interval. Local recurrence = local recurrent versus initially local advanced pancreatic cancer. Distant metastases = distant metastasis before radiotherapy.
